# Simulated Sea Surface Salinity Data from a 1/48° Ocean Model

**DOI:** 10.1038/s41597-024-03314-z

**Published:** 2024-05-23

**Authors:** Frederick M. Bingham, Séverine Fournier, Susannah Brodnitz, Akiko Hayashi, Mikael Kuusela, Elizabeth Westbrook, Karly M. Ulfsax Carlin, Cristina González-Haro, Verónica González-Gambau

**Affiliations:** 1 Center for Marine Science, University of North Carolina Wilmington, Wilmington, NC 28403 USA; 2 Jet Propulsion Laboratory, California Institute of Technology, Pasadena, CA 91109 USA; 3 Department of Statistics and Data Science, Carnegie Mellon University, Pittsburgh, PA 15213 USA; 4 Catlin Engineers & Scientists, Wilmington, NC 28405 USA; 5 Barcelona Expert Center and Institute of Marine Sciences (ICM-CSIC), 08003 Barcelona, Spain

**Keywords:** Physical oceanography, Water resources

## Abstract

To study the validation process for sea surface salinity (SSS) we have generated one year (November 2011- October 2012) of simulated satellite and *in situ* “ground truth” data. This was done using the ECCO (Estimating the Circulation and Climate of the Oceans) 1/48° simulation, the highest resolution global ocean model currently available. The ground tracks of three satellites, Aquarius, SMAP (Soil Moisture Active Passive) and SMOS (Soil Moisture and Ocean Salinity) were extracted and used to sample the model with a gaussian weighting similar to that of the spaceborne sensor ground footprint. This produced simulated level 2 (L2) data. Simulated level 3 (L3) data were then produced by averaging L2 data onto a regular grid. The model was sampled to produce simulated Argo and tropical mooring SSS datasets. The Argo data were combined into a simulated gridded monthly 1° Argo product. The simulated data produced from this effort have been used to study sampling errors, matchups, subfootprint variability and the validation process for SSS at L2 and L3.

## Background & Summary

Sea surface salinity (SSS) has been measured by many different platforms in recent years. Satellite measurements have been made at high spatial and temporal resolution by the ESA SMOS (Soil Moisture and Ocean Salinity), NASA SMAP (Soil Moisture Active Passive) and NASA/SAC-D Aquarius missions^1,2^. Float measurements have been made by the Argo program, which has reached a steady-state population of ~4000 floats^3^. Mooring-based records from the Global Tropical Moored Buoy Array^4–6^ (GTMBA) and other OceanSites^7^ platforms constitute long records of SSS at specific locations. Ships of opportunity (volunteer observing ships or VOS’s) provide high spatial resolution measurements of SSS along commercial ship tracks^8,9^. Process studies such as SPURS-1^10^ (Salinity Processes in the Upper Ocean Research Study - 1) and SPURS-2^11^ have made intensive observations of SSS in limited regions. In addition to direct observations by *in situ* instruments or satellites, there have been many composite or reanalysis products which compile SSS observations into gridded forms to be used for scientific analysis^12–15^. All these observations have been made under consideration of the intimate connection between SSS, the global water cycle and the Earth’s changing climate^1,2,16^.

Retrieval of SSS from satellite observations is complex and challenging in terms of the algorithm and the corrections that need to be applied to the underlying brightness temperature data^17–20^. Individual satellite estimates are noisy, in part due to issues with retrieval, e.g. surface roughness corrections, radio frequency interference, galactic reflection, etc. Most importantly for the present effort, is that satellites measure SSS over a relatively large footprint, typically 35–100 km, due to the long wavelength of the microwave band used for retrieval. Individual level 2 (L2) swath-based observations can be thought of as averaged over the footprint using a gaussian function that approximates the shape of the sensor antenna pattern^20–23^.

Measurement of SSS from *in situ* conductivity and temperature is equally challenging, as sensor drift in the surface ocean over time can degrade measurements if they are not made carefully^3,8,24,25^.

Validation is the process of comparing satellite data to *in situ* ground truth data to ensure the quality of the satellite data^26^. In the case of SSS, there are several types of mismatches in scale and sampling which complicate this process. (1) *In situ* platforms measure over very short spatial scales, i.e. point measurements in the case of Argo floats or moorings, or along-track values in the case of VOS observations. Comparison of those with footprint-averaged values from satellites depends on the amount of variability within the footprint. This type of mismatch is called subfootprint variability^21–23^ or subpixel variability^27^. (2) Salinity satellites sample the true skin layer of the surface, the top ~5 cm^20,21,28^. Most *in situ* platforms, like Argo floats, do not sample the top few cm as they need to turn off their sensors at a few metres depth in order to minimise fouling and prolong their lifetimes^21,29^. There is thus a mismatch between the two measurements depending on the vertical gradient of salinity in the very-near-surface. (3) Satellite validation can be difficult because of temporal aliasing. At L2, this is manifested by the realisation that *in situ* measurements and comparison satellite measurements may not be simultaneous. The intervening variability in the ocean can cause mismatches between satellite and *in situ* data^30^. (4) There is the issue of exactly how to match *in situ* and satellite observations. There are studies^31,32^ that discuss this problem in some detail, describing different ways of accomplishing this, one of which is used^33^ to quantify levels of mismatch error.

All the issues described in the previous paragraph come under the heading of representation (or “representativeness”) error, which is defined as a difference between measured values due to mismatches in sampling or measurement scale^1,27^. This is not really an error in the traditional sense^22^. A data source without any issues in instrumentation or retrieval can still suffer from representation error when compared to some other “perfect” data source because they are not sampling the same environment. There have been many validation studies where satellite data and *in situ* data were compared^19,34–39^. However, all of these studies include both measurement/retrieval error and representation error. Our suspicion is that satellite measurements are better than they may seem as seen in the validation studies just cited because some of the difference between satellite and *in situ* can be attributed to representation errors rather than problems in the retrieval process or instrumental noise. Along similar lines, it has been demonstrated^40^ that the consistency between monthly Aquarius and monthly gridded Argo near surface salinity is comparable to the consistency between the two monthly gridded Argo products in the Tropics and mid-latitudes. *In situ* sampling error is part of the difference in the consistency between Argo gridded products.

The datasets we describe here are being used to understand the size of the representation errors. To do this, we use a high-resolution model to simulate the satellite and *in situ* sampling, so that the validation process can be carried out on the simulated data in the absence of any measurement/retrieval error. In other words, we can generate simulated datasets from a model which mimic as closely as possible the way a satellite samples the SSS field and use it to compare with simulated floats or moorings. Our simulated datasets include satellite SSS at L2 and L3, Argo float SSS data, mooring data for the GTMBA, and a gridded Argo monthly product as described below. We have studied subfootprint variability^22,41^ and matchup errors^32,33^, using the datasets we describe in this paper. Other similar studies are in progress and additional applications can be envisioned.

The datasets presented here were generated as a result of several different studies of satellite SSS errors. Most have been described previously in various places with different purposes^23,30,32,33,41^. Some of these studies make use of the L2 simulation data^23,32,33^, some use the L3 data^41^ and some use the mooring data^30^. The point of the present work is to make these datasets available to the community in a unified way. We have completed some additional evaluation of the L3 and mooring datasets which will be presented in the “Technical Validation” section”. We also introduce some datasets that have not been previously discussed, ones that explore what would happen if the Argo float program were expanded.

The insights gained from using these data can help us to understand the impact of representation error on the retrieval of satellite SSS. We present these datasets with the hope that future researchers can make use of them to gain further knowledge of such errors, and to study what influence changes in the extent of the *in situ* observing system, the configuration of future satellites, or the retrieval process can have. These datasets can also complement the real data provided within the PI-MEP^42^ (Pilot-Mission Exploitation Platform).

## Methods

### The Global Model

The model we use is the 1/48° version of ECCO (Estimating Climate and Circulation in the Ocean), that was originally implemented as a testbed for the SWOT (Surface Water and Ocean Topography) satellite mission^43^. The model is on a latitude-longitude polar cap grid (“LLC4320”) between latitudes 70°S and 57°N. The grid spacing varies from 0.75 km at the far southern extent to 2.3 km at the equator to 1 km at the far north, and the effective spatial resolution is ~8 km^44^. The model run is 15 months long, from which we extracted one year of output (1-November-2011 to 31-October-2012) to produce the simulated products described in this paper. We only looked at SSS, from the top layer of the model, the top metre of the ocean. The model output is hourly for the entire year, though the model time step is shorter than that. The model is free-running: it does not assimilate any ocean data. It is forced with 6-hourly, 0.14°, atmospheric wind and flux fields from the European Center for Medium Range Weather Forecasting (ECMWF). The model uses a constant climatological river discharge^45^. More information about the model, including discussion of forcing, mixing parameterizations, etc. is available^43,46^. The model output that this work is based on can be accessed (https://data.nas.nasa.gov/ecco/data.php?dir=/eccodata/llc_4320). Surface values are also available at the pangeo website (https://catalog.pangeo.io/browse/master/ocean/LLC4320/).

### Satellite Datasets

**Fig. 1:**
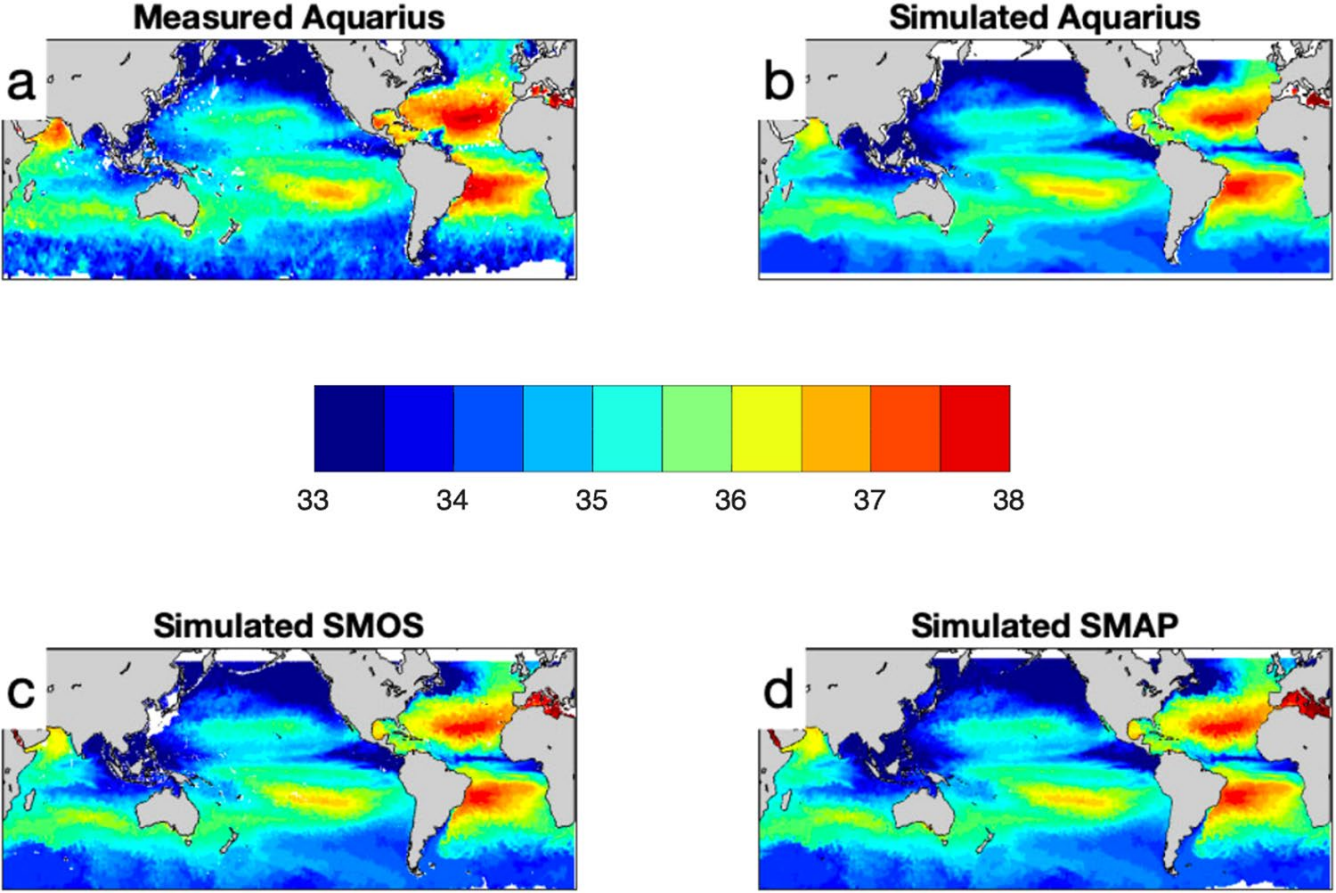
L3 SSS data (in psu) for 4 July 2012. (**a**) L3 measured Aquarius. (**b**) Simulated Aquarius, (**c**) SMOS and (**d**) SMAP. Color scale is in the center.

The three satellites that measure SSS, Aquarius, SMOS and SMAP, were all simulated at L2 and L3. Our intention was to mimic the way that the satellite products are retrieved as closely as possible. For that reason, we took care to make simulated L2 measurements by doing a footprint-average using the specific footprint of each satellite, such as that of Aquarius, whose footprint is ~100 km in diameter^47^. The L2 observations were then combined into L3 products for all three satellites in a manner similar to the way they are made available to researchers. It should be emphasised for these L2 simulated observations that the original L2 data were only used to locate them in time and space. We did not use the observation data themselves, though we do display one set of observational data in Fig. 1a, and some comparisons between real and simulated data in the Validation section of this paper.

### L2 Simulation: Aquarius

We gathered the Aquarius L2 dataset, which goes from August 2011 to June 2015. The original L2 data are available through NASA’s Physical Oceanography Distributed Active Archive (PO.DAAC)^48^. Aquarius sampled in a 3-beam pattern^2,47,49^, with L2 observations every 1.44 s along the track of the satellite. The dataset we used in this study was the Aquarius L2 data collected during the one-year period for which we had model output. Including only those points whose land fraction value was less than 0.5%, this amounts to about 31 million L2 observations. The L2 simulation values were determined at the closest hourly time step of the model. The process of generating simulated L2 observations is described in detail^23^.

The Aquarius L2 simulated data include 2 versions with gaussian noise added, with 0.1 and 0.2 standard deviation to test matchup criteria^32,33^.

### L2 Simulation: SMAP

**Fig. 2:**
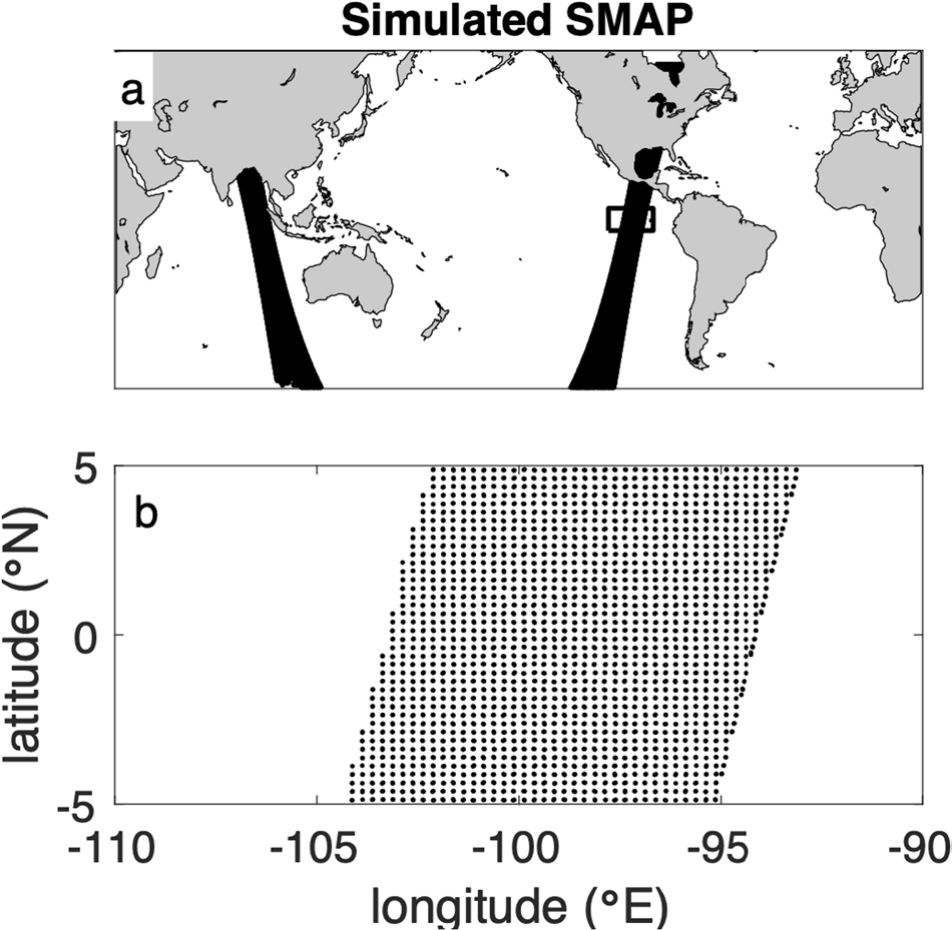
An illustration of the 1/4° sampling of SMAP. (**a**) One example swath. A box outlines the area displayed in panel b. (**b**) Individual L2 observation locations.

For SMAP, the L2 observations are taken on a ¼° swath-based grid centered along the nadir point of the satellite track (Fig. 2). SMAP has a rotating, scanning antenna that samples in a trochoidal pattern over the Earth’s surface^2^, with individual snapshots averaged to form these gridded values. Each simulated L2 observation is formed by weighted average of the model onto the ¼° grid at a spatial resolution of ~40 km^20^. The original L2 observation data are available through PO.DAAC (10.5067/SMP40-2SOCS).

SMAP was launched in early 2015, so the time period of SMAP does not coincide with that of the model output. To finesse this issue, we took the times of the SMAP L2 observations between 1 November 2016 and 31 October 2017 and subtracted 5 years. So the SMAP simulated dataset we present is as if the SMAP satellite flew over the ocean and sampled in 2011-2012^32^. There are a total of about 142 million simulated SMAP L2 observations.

### L2 Simulation: SMOS

For SMOS, we started with the L2 v700 SMOS SSS product produced and distributed by ESA - again just the times and locations. The data are available through the ESA SMOS Online Dissemination Service (https://esatellus.service-now.com/). The SMOS footprint diameter varies from 35 km at nadir to 70 km at the maximum incidence angle^50^. We made use of SMOS-generated L2 values on about a 6° wide grid relative to the satellite track (314 km to either side of the nadir). We used the same gaussian-weighted average to get the L2 simulated values for SMOS, but with a variable footprint size. The weights depend on the radiometric accuracy of each gridpoint^41^. There are a total of about 308 million simulated SMOS L2 observations.

### L3 Simulation

Simulated L3 SMAP and Aquarius data were produced by smoothing or averaging, using the L2 values as input^41^. The SMAP and SMOS L3 data were produced on a regular 1/4° grid and the Aquarius data on a 1° grid.

For Aquarius for each 1° grid cell, all L2 observations within a given time period were smoothed using a local polynomial fit^51^ to produce the L3 estimates - 7-day running mean and monthly.

For SMAP for each 1/4° grid cell, all L2 observations within the cell within the given time period (8-day or monthly) were simply averaged to produce the L3 estimates - 8-day running mean and monthly^20^.

Generation of the simulated SMOS L3 values was a little more complicated than for the other satellites^41^. We averaged L2 values within each 1/4° grid cell over 9-day and monthly time periods using a weight that depends on the radiometric accuracy^50^. That is, the SMOS simulated value at each grid cell is the weighted average of all L2 values that fall within that cell using weights computed from the radiometric accuracy, the more accurate the measurement the higher the weight^41^.

### *In situ* data

We created simulated *in situ* datasets from Argo floats and from moorings. The original Argo and mooring SSS data were (mostly) not used in this study, just the times and/or locations of the samples.

### Argo Data

**Fig. 3:**
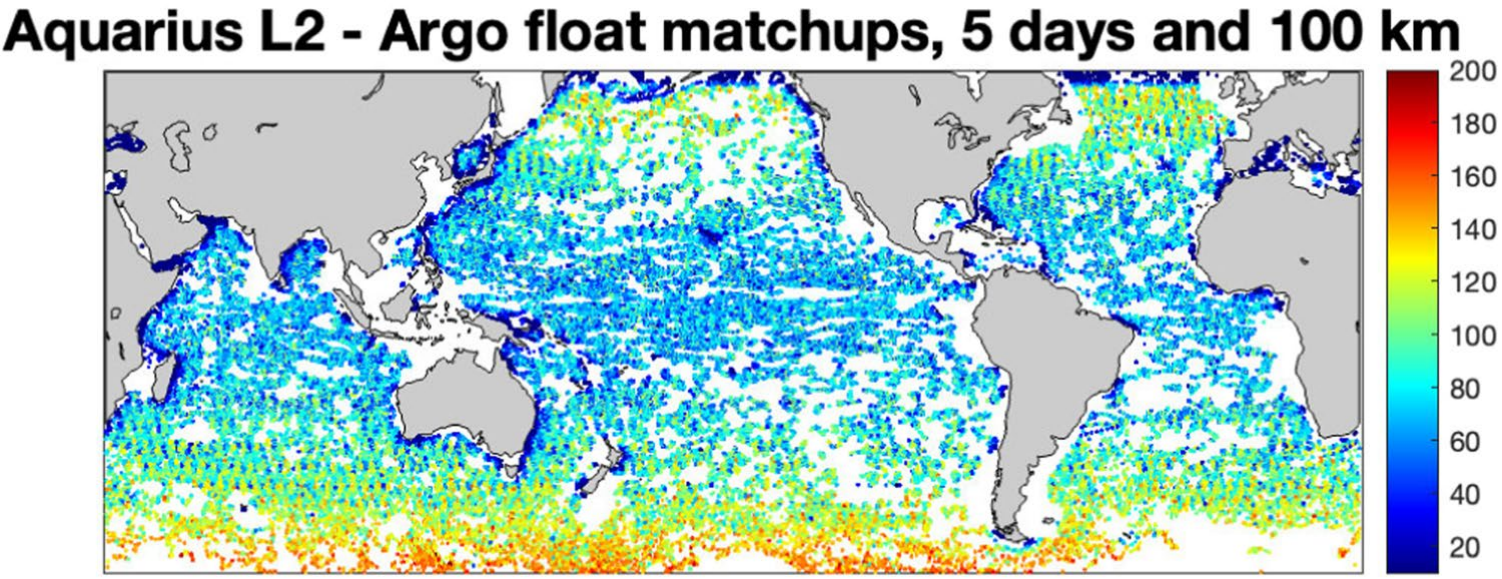
Locations of the Argo floats observations used to generate the simulated data. Color (scale at right) indicates the number of Aquarius L2 observations located within 5 days and 100 km of each float observation.

We gathered a dataset of ~101,000 float surfacings over the one-year study period^52^. That is, we noted the dates, times and locations when each float came to the surface, found the nearest model estimate in space and time, and compiled the set of these into a simulated Argo dataset. We used only float profiles with a quality flag of “1 - Good Data” and where the topmost measurement was above 10 m depth. A picture of the float distribution over the year can be found in Fig. 3. It should be noted that the model data are surface values, the top 1 metre. As stated above, Argo floats typically stop sampling at ~5 metres depth^29^. Thus, the mismatch due to the differences in sampling at the surface are almost completely eliminated in the data products documented in this paper.

**Fig. 4:**
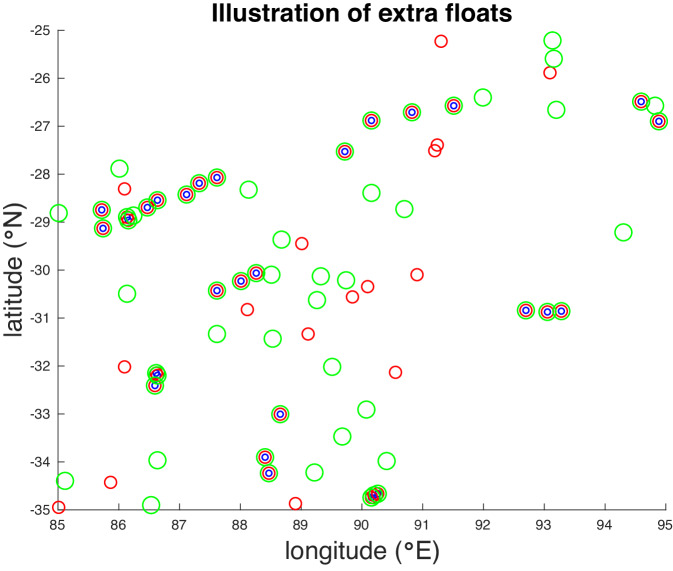
Positions of float observations for July 2012 for a 10°X10° area of the South Indian Ocean. Blue circles: Original Argo data. Red circles: 50% extra floats. Green circles: 100% extra floats.

In addition, we generated datasets with extra floats. That is, we took each 1° area in each month, found the number of float surfacings in that area, and then randomly selected additional (for example, 50% more) locations in the same area and time period to simulate extra floats. The purpose of this extra simulated sampling was to study time aliasing issues related to sampling by evaluating the impact of the frequency of sampling on the sampling error in the *in situ* gridded products. Our “added float” datasets include ones with 10, 20, …,100% extra floats (Fig. 4).

We created a simulated surface version of a gridded Argo dataset^12^. The original is an optimally-interpolated 4-dimensional monthly product on a 1°X1° grid. It is one of several such gridded products commonly used to validate satellite SSS data^40^. The documentation and codes used to compute the Argo-based gridded product are available^53^. More detail on how this product was processed to make the current product are available^41^. We created gridded products on 1° and 1/2° grids, and also ones using the range of extra float input datasets described in the previous paragraph.

There is an important difference between this simulated dataset and the satellite datasets we have produced. That is, due to the method by which the dataset is produced, the simulated data are not completely independent of the real Argo data that underlie the real original dataset^41^. Our output product includes versions with one iteration or two of the gridding process. The two-iteration version is closer to being independent of the underlying real Argo data, but not completely.

### Mooring Data

The GTMBA is a vast network of buoys in the tropical oceans. It was originally set up in the mid-1980s to measure El Niño-related variability in the tropical Pacific^4,6^ and has since been expanded to the Atlantic^54^ and Indian^5^ Ocean basins. This dataset has been used by many authors to validate satellite SSS data^35–37,55,56^. There are 131 moorings in the historical record, many of which have large gaps, or are no longer operating. These moorings measure salinity at ~1 m depth^25^. Some of the moorings were not operating during the 2011-2012 study period. Despite this, we created a simulated mooring dataset by finding the nearest grid node to each mooring location, and extracting samples at the hourly time-step of the model. (The real mooring data are also mostly sampled hourly.) These simulated mooring data were used^30^ to examine short-term variability in comparison with real mooring and satellite data. It was found that short-term (<~7 days) variability from the model is comparable to that from the real mooring data, but a little smaller, with a median representation error of 0.09 for the real mooring data vs. 0.07 for the model.

### ECCO Grids

Another product we produced was averages of the model within grid boxes. All of these products were simple averages of all data within the given box^41^. The idea was to simulate the average ocean over various space and time scales without the extra complication of footprint averaging and combination of L2 values described above. This can be thought of as a “ground truth” for simulated L3 fields. These grid-box averages are available on 9-day (¼°X¼°), 8-day (¼°X¼°), 7-day (1°X1°), and monthly (1°X1° and ¼°X¼°) time scales.

## Data Records

The simulated SSS dataset is available at the University of North Carolina (UNC) Dataverse at the locations cited below^57–62^. The individual files we have generated are all in netCDF format and packaged together in compressed (.zip) form (except for the simulated mooring data, which are not compressed). The data reside in the following directory structure:

ECCO grids^57^

                   7-day

                                   1 × 1

                   8-day

                                   qXq (1/4° × 1/4°)

                   9-day

                                   q × q

                   monthly

                                   q × q

                                   1 × 1

Simulated Aquarius^58^

               SimulatedAquariusL2

               SimulatedAquariusL3

                             7-day

                             monthly

Simulated Argo^59^

               SimulatedArgoFloats

                              SimulatedArgoFloatsOriginal

                              SimulatedArgoFloats10PercentExtra

                              SimulatedArgoFloats20PercentExtra

                              …

                              SimulatedArgoFloats100PercentExtra

               SimulatedArgoL3

                             HalfDegreeGrids

                                              SimulatedArgoL31OriginalHalfDegree

                                              SimulatedArgoL350PercentExtraHalfDegree

                                              SimulatedArgoL3100PercentExtraHalfDegree

                             OneDegreeGrids

                                              SimulatedArgoL3Original

                                              SimulatedArgoL310PercentExtra

                                              SimulatedArgoL320PercentExtra

                                              …

                                              SimulatedArgoL3100PercentExtra

                             SimulatedArgoL3OneIteration

Simulated Moorings^60^

Simulated SMAP^61^

               SimulatedSMAPRSSL240km

               SimulatedSMAPL3

                             8day

                             monthly

Simulated SMOS^62^

               SimulatedL2SMOS

               SimulatedL3SMOS

                             9day

                             monthly

The simulated satellite datasets, Aquarius, SMAP and SMOS, have L2 and L3 versions. The L2 are individual observations arranged as vectors, with time, latitude, longitude and simulated SSS. The L3 versions are gridded files, with vector time, latitude and longitude values and matrix simulated SSS values dimensioned by latitude, longitude and time.

The ECCO grid dataset is structured the same as the L3 satellite datasets.

The simulated Argo dataset also has L2 and L3 versions, structured in the same way as the satellite datasets.

The simulated mooring dataset has vector time, latitude and longitude describing the locations of the virtual moorings. The SSS from the moorings is a matrix with dimension (#moorings X time).

## Technical Validation

A comparison of one week of actual Aquarius observations (panel a) and the same week of simulated values (panel b) is shown in Fig. 1. The Aquarius values are noisier, especially at high latitudes. Also of note is the lack of a distinctive Amazon River plume in the simulated data, due to the use of climatological river discharge data as river forcing as described above. Otherwise, the patterns of SSS at large scales are similar between the figures^33^. In any case, the point of the simulated dataset is not to simulate the actual SSS, but to use the model to test validation and matchup procedures. As the model is free-running and does not assimilate real ocean data, one would not necessarily expect detailed correspondence between model and observations.

**Fig. 5:**
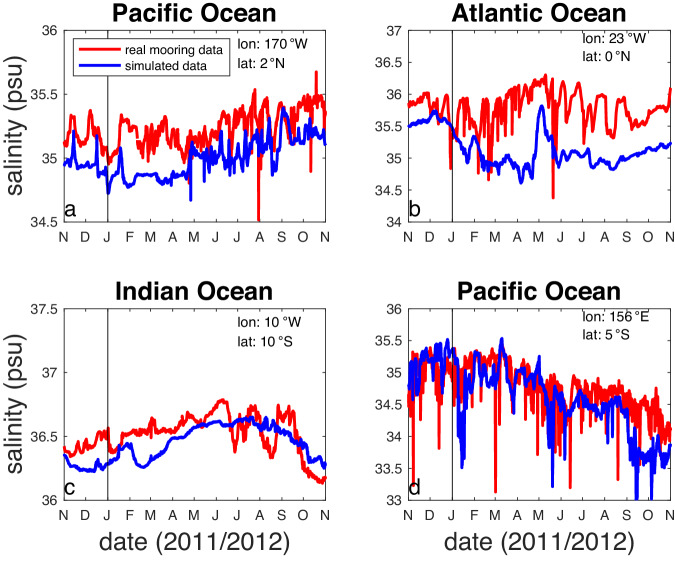
Some example records of real mooring data (red curves) and model data extracted from the mooring location (blue curves). Locations for the moorings are noted in each panel (**a**-**d**).

A typical comparison of real and simulated mooring data at a sample of moorings is included in Fig. 5. The real mooring data have more very short time scale low salinity spikes, likely due to rain events that are not well-captured by the reanalysis-based freshwater forcing imposed on the model^30^.

**Fig. 6:**
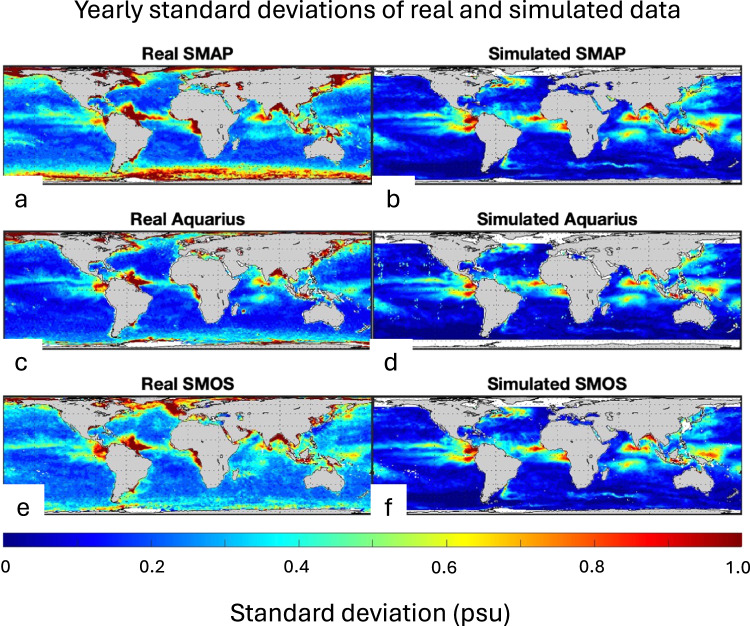
Standard deviation of SSS over the one-year evaluation period. For (**a**) Aquarius, (**b**) simulated Aquarius, (**c**) SMAP, (**d**) simulated SMAP, (**e**) SMOS, (**f**) simulated SMOS.

**Fig. 7:**
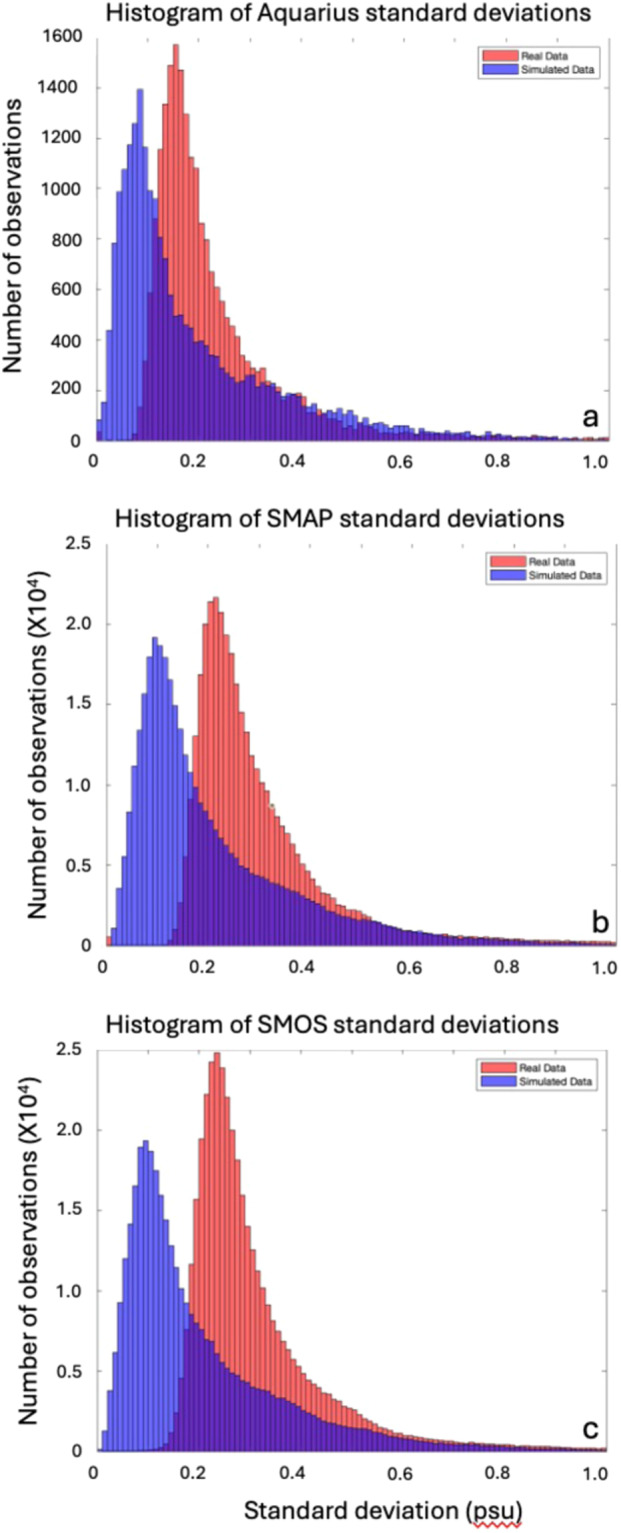
Histograms of the data presented in Fig. 6, but limited to latitudes 45°S to 45°N. The blue (red) curves summarise the data presented in the right-hand (left-hand) column of Fig. 6. The y-axis represents the number of 1° or ¼° grid cells with the given standard deviation. (**a**) Aquarius. (**b**) SMAP. (**c**) SMOS.

In general, it appears that the model SSS is less variable than the real SSS (Figs 5–7). We speculate that this is a result of the 6-hourly, 0.14° freshwater forcing that the model experiences. The real freshwater forcing over the ocean likely has smaller space and time scales than that^63,64^. The impact of small-scale spatial variance of SSS on ocean dynamics is an area that deserves more attention, but cannot be completely resolved using the datasets described here. It is clear that the treatment of river discharge in the model impacts regions surrounding major river plumes, the Amazon and Mississippi most notably.

A few areas are more variable in the model (Fig. 6). These include the eastern tropical Pacific, the western equatorial Pacific and the tropical South Indian Ocean. The modes of the standard deviation histograms are higher for the real relative to the simulated data (Fig. 7). This diminished variability in the model makes it less than ideal for evaluating representation error in SSS data, but at this point it may be the best we can do. No real *in situ* dataset can provide the type of spatial and temporal coverage required to adequately evaluate representation error, though some may come close in limited temporal or spatial domains^22,30,63^.

One issue that comes up when thinking about the data we present here has to do with the stratification of the upper few metres of the ocean, and the ability to represent it within the ECCO model. It is well known that low salinity events can be confined to the top metre or two of the ocean^9,21^. As discussed above, Argo floats usually turn their pump off and stop sampling on their ascent at ~5 m depth, and that this can cause some mismatch between satellite and *in situ* sampling^29^. The Argo float dataset we have described is taken from the top metre of the model, and thus does not necessarily represent actual Argo float samples as they occur in the real world. It is not clear however, that the ECCO model represents the detailed stratification of the top metre correctly. How rain–formed fresh lenses are mixed into the interior is an area of active research^65–69^. Figure 5 compares the top metre of the model with mooring data from instruments at 1 m depth. It shows that the model has less variability than the real mooring data and does not have the same spikiness or influence from shallow low-salinity lenses. How this would impact our determinations of representation error is an area for potential future study.

## Usage Notes

These data are available under a Creative Commons CC BY 4.0 license (https://creativecommons.org/licenses/by/4.0/).

## Data Availability

The code for doing the computations described in this paper is available at the UNC dataverse sites given in the “Data Records” section above.
